# Scaling up and scaling down: Improvisational handling of critical work practices during the COVID-19 pandemic

**DOI:** 10.1177/13505076221137980

**Published:** 2022-12-08

**Authors:** Martina Berglund, Ulrika Harlin, Mattias Elg, Andreas Wallo

**Affiliations:** Linköping University, Sweden; RISE Research Institutes of Sweden, Sweden; Linköping University, Sweden

**Keywords:** Adaptive learning, developmental learning, organisational change, organisational learning, qualitative, responsiveness

## Abstract

The aim of this article is to explore improvisational handling of critical work practices during the COVID-19 pandemic and interpret these practices from a learning perspective. Based on an interview study with representatives of private, public and intermediary organisations, the study identified three different types of improvisational handling as responses to the pandemic crisis involving ‘scaling up’ and ‘scaling down’ critical work practices. By ‘scaling up’ and ‘scaling down’, we refer to practices for which, due to the pandemic, it has been imperative to urgently scale up an existing operational process or develop a new process, and alternatively extensively scale down or cease an existing process. The types of improvisational handling differed depending on the discretion of involved actors in terms of the extent to which the tasks, methods and/or results were given beforehand. These types of improvisational handling resulted in temporary solutions that may become permanent after the pandemic. The framework and model proposed in the article can be used as a tool to analyse and learn from the changes in work practices that have been set in motion during the pandemic. Such learning may improve the ability to cope with future extensive crises and other rapid change situations.

## Introduction

In the wake of the COVID-19 pandemic, the capacity for learning and innovation has been identified as a crucial component in strategies for organisational change and human resource development (Dirani et al., 2020; Karjalainen et al., 2022; Laitinen and Ihalainen, 2022; McLean and Jiantreerangkoo, 2020; Yawson, 2020). Because of the rapid progress of the pandemic, many organisations have been forced to respond swiftly to the changing circumstances. During the crisis, not only individual work tasks but also the entire work situation and collaborations between professional groups and departments have been affected. These new situations have enabled different types of improvisations in organisations, both in terms of the behaviours of individual managers and employees and in terms of changes made to organisational procedures and work practices. Here, ‘improvisation’ is defined as purposeful action in response to unexpected interruption using available resources and without established routines (building on Crossan et al., 2005; Cunha et al., 1999; Hadida et al., 2015; Miner et al., 2001), while ‘improvisational handling’ refers to different types of actions and approaches within organisations related to critical work practices during the COVID-19 pandemic. Furthermore, in fast-changing organisational environments, multiple tensions and contradictions may arise requiring attention to the impact of global, dynamic and contradictory demands that may occur (Smith and Lewis, 2011), and therefore also need to be considered during improvisational handling. Learning during such crises plays a vital role in facilitating organisational transformation, however, there is a need to take power relations and group interactions into account when exploring long-term learning outcomes of improvisations (Meisiek and Stanway, 2022).

Previous studies of management and organisational learning during severe economic crises show that organisations are more likely to succeed if they can manage their day-to-day operations and at the same time, adapt to major changes (Wallo et al., 2012). This includes the possibility to scale up and scale down operations. However, even if organisations have previously needed to respond through improvisation, these improvisations have been situated, temporary and not premeditated (Crossan et al., 2005) and used mainly as ways to increase the capacity to meet the unexpected (Weick, 1998). In this article, we argue that the COVID-19 pandemic represents an unprecedented situation when the management of both day-to-day operations and the adaptation to major changes have generated a palette of different means for improvisation and organisational learning. Looking at improvisation as a source of learning in organisations (Cunha et al., 1999; Miner et al., 2001; Vendelø, 2009), learn-by-doing activity (Rerup, 2001) and purposeful action using available resources in the absence of a plan (Hadida et al., 2015), the pandemic offers potential for organisational development beyond dealing with the actual crisis. Although there are some initial studies on improvisation related to the COVID-19 pandemic, for example, highlighting different strategies for handling new demands for change (Cox et al., 2021), reflective practice to support the process of rapid improvisation (Bryson and Andres, 2020) and the need to loosen control to facilitate organisational improvisation (Lloyd-Smith, 2020), this potential for organisational development warrants further investigation. In addition, there appears to be a lack of previous empirical research on improvisation and learning in organisations (Cunha et al., 1999; Easterby-Smith, 1997; Kamoche et al., 2003a, 2003b; Meisiek and Stanway, 2022; Vendelø, 2009; Vince et al., 2002).

Thus, research is needed to investigate how organisations have managed critical work practices brought on by the COVID-19 pandemic by means of different types of improvisational handling, but also how learning can occur during these improvisations. The aim of this article is therefore to explore improvisational handling of ‘scaling up’ and ‘scaling down’ critical work practices during the pandemic and interpret these practices from a learning perspective. By ‘scaling up’ and ‘scaling down’, we refer to critical work practices, for which, due to the pandemic, it has been imperative to urgently scale up an existing operational process or develop a new process, and alternatively extensively scale down or cease with an existing process.

The empirical setting of the study is in a Swedish context, where on 1 February 2020, the Swedish Government classified COVID-19 as a disease that constituted a danger to society, which opened possibilities of extraordinary communicable disease control measures. An important starting point for government decisions and actions was careful considerations of the expert knowledge contributed by government agencies such as the Public Health Agency of Sweden, an expert authority with responsibility for public health issues at a national level.^1^ Furthermore, crisis management in Sweden is built on the principle of responsibility, meaning that any party responsible for a particular activity under normal circumstances is also responsible for that activity during a crisis. Based on these recommendations, in March 2020, the Swedish Government declared several measures and actions concerning businesses and organisations to limit the spread of infection in the country, ensure availability of health and medical resources, limit the impact on critical services and alleviate the impact on people and businesses, saving people’s jobs and livelihood. Several restrictions and recommendations were carried out. For instance, on 16 March, it was recommended that employees with work tasks possible to be carried out remotely should do this from home.^2^ This article, therefore, also addresses the perspectives of selected management and expertise representatives (from, for example, trade union organisations) with a national role for both private and public sectors.

The findings of our study contribute empirically based knowledge of improvisation and learning in organisations during a severe crisis, in particular, concerning critical work practices during the early phases of the COVID-19 pandemic. By focusing on the need for improvisational handling in the swift changes during the pandemic, this study also contributes knowledge that may increase preparedness for future crises, or other major changes and transformations in organisations. We also elaborate and extend theorisation on how the concepts of improvisation can be related to theories on learning in organisations.

We present our investigation in four stages. First, we discuss the theoretical underpinnings of the article, which comprise key concepts and theories within the fields of improvisation and organisational learning. Second, the critical incident methodology employed in the study is outlined. Third, we present and discuss our findings. In the fourth and final part, we discuss the study’s significance, its implications for research and practice, and its limitations before we conclude the article.

## Theoretical background

### Improvisation

From being considered as a disturbance in organisational equilibrium (March and Simon, 1958) or deficiency in organisational design (Mackenzie, 1986), improvisation in organisations has become increasingly interesting since the mid 1990s (Vendelø, 2009). Crossan et al. (2005) argue that a complementary view of improvisation as undesired potential variation in organisations ‘where everything unpredictable should be removed and replaced by rationalised certainty’ as stated by Cunha et al. (1999: 513), is to consider improvisation an integrated part of organising operations to respond to the emergent and unexpected deviations from standard routines that occur constantly in everyday work.

With respect to improvisation, several characteristics have been described, for example, that the response to the unexpected emerges from within or outside the organisation (Hadida et al., 2015) and that improvisations occur when people are overwhelmed by the world and therefore need to regard it differently (Ciborra, 1999). Several researchers also state that improvisation occurs when the organisation needs to be responsive to the environment (Hatch, 1998) and cannot apply pre-approved routines, actions and solutions (Cunha et al., 1999; Hadida et al., 2015; Hatch, 1998; Moorman and Miner, 1998; Weick, 1993). Thus, thinking and acting are co-evolving in any improvisational activity, where the meaning of actions is evaluated in retrospect (Hadida et al., 2015). Although improvisation is situated within a context with an overall purpose, intuition influences the actions in spontaneous ways (Crossan and Sorrenti, 1997). Cunha et al. (1999) further state that improvisation is characterised by having a purpose, being extemporaneous and occurring during action. Hadida et al. (2015) state that this action is taken in response to an unexpected interruption or change of activity. Another important characteristic is that the action draws on available resources (Cunha et al., 1999). Research shows that time pressure and transforming contexts constitute external triggers of improvisation (Augier et al., 2001; Miner et al., 2001). These aspects in combination with a need to quickly solve problems for which no established routines exist may also trigger improvisation (Crossan et al., 2005; Miner et al., 2001). In line with the above, we thus adhere to the definition of improvisation as purposeful action in response to unexpected interruption using available resources and without established routines.

As a response to the varied collection of research on improvisation in organisations, Hadida et al. (2015) have proposed a consolidating framework based on the degrees and levels of improvisation. The degree of organisational improvisation is categorised along a continuum from ‘minor’ (related to performing an existing task in a different way), ‘bounded’ (referring to improvising a different task towards the same outcome) and ‘structural’ (improvising a different task towards a new outcome) improvisation. The level of organisational improvisation is similarly divided into three types, referring to the improvisation taking place within an individual, between a few employees, or organisational. In the latter case, the entire organisation is considered to have the ability to improvise and there are formal structures or practices to support improvisation (Hadida et al., 2015). Although they claim that their typology was developed through a systematic review of the literature, Ciuchta et al. (2021) point out that the work of Hadida et al. is a narrative review focused on developing a framework rather than ‘providing a comprehensive and systematic review of antecedents, outcomes, and processes associated with the OI construct’ (Ciuchta et al., 2021: 293). Furthermore, Hadjimichael and Tsoukas (2022) have identified a gap in traditional approaches, such as the work of Hadida et al. (2015), claiming that research is lacking regarding values and moral dimensions in organisational improvisation.

### Organisational learning

Although considered a neglected area within organisational learning (Crossan and Sorrenti, 1997), research shows that improvisation is related to learning (Crossan and Sorrenti, 1997; Miner et al., 2001; Vendelø, 2009) as a distinct activity (Bergh and Lim, 2008; Cunha et al., 1999; Hmieleski et al., 2013; Xiang et al., 2020). Cunha et al. (1999) further note that organisations engaged in improvisation learn how to improvise. They also learn when routinising what first has been improvised. Finally, during the improvisational action, they learn about themselves and their environment. Another identified learning potential during improvisations lies in the formalisation or routinising of the improvisations, which is part of organisational learning. Organisational learning is here defined as changes in organisational practices, such as procedures, structures, routines, technologies and policies, which are facilitated by processes of individual and group-based learning (Wallo et al., 2012). Thus, learning at the individual and group levels can be seen as a necessary but insufficient condition for organisations to increase their capacity to handle different situations, problems and challenges (Ellström, 2001).

A well-known framework for understanding organisational learning is the so-called ‘4I framework’, which consists of the following four sub-processes: (1) intuiting, (2) interpreting, (3) integrating and (4) institutionalising. This framework aims to capture the complex, continuous interplay between the individual, group and organisational levels (Crossan et al., 1999). According to this framework, learning begins at the individual level when people realise and articulate something new in their work (intuiting). The individual forms explanations and shares them by discussing them with colleagues (interpreting). Gradually, a partially new knowledge base is developed, which is incorporated into the practices of working groups (integrating). In the final stage (institutionalising), the new insights have been established at the organisational level, which is then fed back to individuals and groups throughout the organisation as policies, routines and strategies. The first three processes are relatively common in organisations, but institutionalisation is less common and takes more time to develop (Crossan et al., 1999). According to Crossan et al. (1999), it is also important to increase the understanding of tensions between the exploration of new knowledge in a feed-forward process and the exploitation of these skills in a feedback process, as illustrated in the ‘4I framework’. Thus, there is a need to further investigate how to deal with these tensions in practice. This can be compared to literature on power and paradox studies that highlight the need of understanding multiple tensions that arise and how to manage complexity and competing demands simultaneously (Smith and Lewis, 2011) in a fast-changing and competitive environment. Thus, as recently explored in studies during the COVID-19 pandemic, there is a need to possess an ability to constantly consider different types of paradoxes that may occur in new contexts when improvising (Vera and Crossan, 2022), as well as a need for deeper understanding of the ‘cognitive and social dynamics’ that can arise between individual improvisation and collective actions (Hadjimichael and Tsoukas, 2022).

### Levels of learning

How, then, can we understand the nature of the learning in the model proposed by Crossan et al. (1999)? In this article, we draw on Ellström’s (2001, 2006) identification of four different levels of learning that exist in work situations based on the discretion of the task to be carried out, the methods to be used and the expected result.

The first level, reproductive learning, comprises actions that are routinised and performed without conscious control. This is related to tacit knowledge (Polyani, 1966) and skill development in frequently recurring actions. Since the task, method and expected results are predetermined, there is no room for improvisation, and this level of learning will, therefore, not be further discussed in the article. The next level of learning proposed by Ellström (2001, 2006) is productive learning (Engeström, 1987), which is divided into rule-oriented learning and goal-oriented learning. For both levels of learning, the task is predetermined. However, for rule-oriented learning, there is discretion regarding the evaluation of the result, while there is discretion regarding the choice of methods for goal-oriented learning. In the latter, the individual faces a situation in which predetermined routines cannot be applied. The fourth level of learning is creative-oriented learning, where the individual must use his or her own authority to define the task, choose methods and evaluate the results. None of these aspects (task, method and results) are predetermined, implying that alternatives need to be considered and established norms, structures and practices can be questioned. As there are combinations of discretion in tasks, methods and/or results within rule-oriented and goal-oriented learning as well as in creative-oriented learning, there is also room for improvisation. According to Martin et al. (2018), this implies that creative learning seems to be reliant on active engagement and competence among organisational participants.

### Conceptual framework – improvisation and learning combined

By integrating Ellström’s (2001, 2006) proposed levels of learning in terms of discretion of tasks, methods and results with an interpretation of the continuum of degrees of improvisation proposed by Hadida et al. (2015), we propose a conceptual framework that can be used to analyse improvisational handling and responses in critical work practices at the individual, collective or organisational levels (Table 1). Thus, the conceptual framework highlights the need to understand the role of tasks, methods and expected results in improvisation and learning. Complexity in improvisation resides in the different combinations of how we understand tasks, methods and expected results or possible outcomes. In line with Martin et al. (2018), we also argue that learning is not a hierarchical phenomenon in the sense that ‘higher-level’ learning processes assume superiority over ‘lower-level’ learning processes. Rather, we adopt the view that different contexts and settings induce different learning processes, sometimes even simultaneously.

**Table 1.** Conceptual framework – improvisational handling types and levels of learning.

	Improvisational handling type A	Improvisational handling type B	Improvisational handling type C
**Degree of organisational improvisation** ^a^	Minor Performing an existing task in a different way	Bounded Improvising a different task towards the same outcome	Structural Improvising a different task towards a new outcome
**Discretion** Task Method Result (Expected)	Low One of the aspects is ‘not given’ beforehand (either method^a^ or result^b^)	Medium Two of the aspects are ‘not given’ beforehand (either task & method^a^ or method & result^b^)	High None of the three aspects (task, method and result) are given beforehand
**Levels of learning** ^b^	Rule-oriented learning	Goal-oriented learning	Creative-oriented learning

Source: Adapted from Hadida et al. (2015)^a^ and Ellström, (2001, 2006)^b^

In the framework, improvisational handling type A involves rule-oriented learning (Ellström, 2001, 2006) and can be expected to generate or respond to minor changes in contexts where the discretion is low, that is, when the task, method or result are mostly ‘given’. An example of improvisational handling type A can be performing the same task using a different type of procedure to reach the same result. Improvisational handling type B instead mainly occurs when the discretion is at a medium level, that is, when two out of three of the aspects task, method or result are ‘not given’. This type of improvisation corresponds to bounded improvisation (Hadida et al., 2015) and entails goal-oriented learning (Ellström, 2001, 2006). With reference to Hadida et al.’s work, one example could be when an intended result is clear and pursued through a modified task and methods other than those used before. Finally, improvisational handling type C, which bears a resemblance to the notion of structural improvisation (Hadida et al., 2015), is prevalent when the discretion level is high in task, method as well as result. One example can be an identified need for a major transformation within your own organisation’s business domain, but there is limited knowledge of how this can be reached. There is a lack of clear tasks and established methods which requires a new mind-set and new approaches. As a result, the outcome is not given beforehand. This gives room for creative-oriented learning (Ellström, 2001, 2006) that is expansive (Engeström, 1987) and encompasses critical reflection, experimentation and risk-taking (Wallo et al., 2022).

The three types of improvisational handling, and corresponding levels of learning, can occur at all levels in the organisational learning model that was described previously (Crossan et al., 1999). In line with this, it is assumed that during major change situations in organisations, improvisation at the organisational level is mediated through individual and collective improvisations. Thus, similar to Crossan et al.’s (1999) notion of organisational learning, individual and collective improvisations are viewed as a necessary, but not sufficient condition for organisational improvisations. An increasing challenge raised by Smith and Lewis (2011) is the need of constant reflection and a holistic understanding of organisational tensions, and development of management strategies to manage an increasingly complex reality in organisations. Thus, they highlight the need for cyclical and dynamic management approaches in fast-changing organisations to foster creativity, learning, flexibility and resilience.

## Methods

The research setting for the project comprises organisations in the Swedish labour market, which is characterised by a high density of powerful trade unions and a high presence of collective agreements between unions and employers (Wallo and Kock, 2018). In Sweden, there is also legislation with far-reaching consequences for employers and employees. Most notable are the Co-determination Act and the Act on Security of Employment (Mabon, 1995). Hence, the Swedish context of the labour market is commonly described as the Nordic model, where employee and employer parties jointly participate and collaborate in major change processes, with a shared vision and mission to create solutions and value for people and the organisations (Garmann-Johnsen et al., 2018). Thus, the respondents were purposefully selected regarding their strategic function within public and private organisations, as well as with respondents with a national intermediary role in private and public sectors in Sweden. This enabled capturing knowledge of both sectors and the particular organisations with insights into the handling of the pandemic. A balance was sought between the interview groups, and in total, the empirical material comprised 29 interviews and 31 respondents; 28 individual interviews and one focus group interview including three people (see Table 2).

**Table 2.** Overview of respondents.

Group	Interviews	Respondents’ roles and functions
Public sector	Nine individual interviews	Top management and expertise within regional health care and municipalities (directors, human resource managers)
Private sector	Nine individual interviews	Top management within industrial development (global senior advisors, research&development directors, business area managers within industrial manufacturing sectors such as automotive and medical industry)
Intermediaries for public and private sectors	10 individual interviews one focus group interview (three people)	Social partners from employee and employer organisations (trade unions) representing enterprises, companies, public organisations and workplaces

The effort to capture the situational nature of the studied phenomena in this study, that is, the fact that the study was carried out during the early phase of the COVID-19 pandemic, necessitated a methodology that was able to capture the interplay between individual agency and the structural conditioning of the context (Archer, 1995; Danermark et al., 2019). For that reason, we chose to conduct semi-structured interviews with inspiration from the Critical Incident Technique (Flanagan, 1954) as our primary approach for exploring organisational learning in relation to improvisations during the pandemic.

In the interviews, respondents were asked to reflect on the impact of the COVID-19 pandemic in organisations and at their workplaces with a focus on ‘scaling up’ and ‘scaling down’ changes. These changes were categorised in this study as critical work practices, thus initiating work tasks to achieve the desired result by using different methods. However, these tasks had different prerequisites regarding the expected results. Findings on methods, together with the respondents’ reflections on uncertainties, challenges and possible long-term effects ‘post-pandemic’, enabled analyses according to different levels of learning (rule-oriented, goal-oriented and creative-oriented learning).

The interviews were conducted digitally using Microsoft Teams or Zoom. Although the interviews were face-to-face, it was important to allow time for interviewee questions to establish and maintain a positive relation (Miles and Huberman, 1994). The interviews lasted on average 60 minutes and were recorded and transcribed verbatim, in total covering 20 hours.

The data from the respondent groups ‘public sector’ and ‘private sector’ were related to their organisation. The data from the group ‘intermediaries’ were related to their organisation, as well as to private and public sectors in general.

The respondents contributed with data from an individual and organisational view, where the level of analysis had an organisational perspective. This was in line with the earlier described view of understanding improvisation at the organisational level as being mediated through individual and collective improvisations. The analysis of the interviews was abductive, that is, it was not theoretically driven, but rather alternated between inductive interpretations and tentative links to previous research and theory. The analysis was conducted in multiple steps, following procedures outlined by Miles and Huberman (1994).

The *first step* was carried out to identify major changes ‘scaling up’ and ‘scaling down’ during the pandemic, categorised as critical work practices, and to find examples in different domains (public sector, private sector or within intermediaries for their organisations). Thus, these critical work practices inductively generated terms that were used as metaphors for processes in which improvisational handling occurred. The *second step* consisted of selecting illustrative examples of work tasks related to these critical work practices, methods (approaches, actions) and expected results. The assessment of tasks, methods and expected results included analyses whether there were ‘given’ or ‘not given’ prerequisites or conditions related to these aspects. Table 3 provides a description that served as a guideline for the categorisation.

**Table 3.** Assessment of the aspects task, method and result into ‘given’ or ‘not given’.

Aspect	‘Given’	‘Not given’
**Task**	Clear tasks, explicit problems	Unclear tasks, complex problems
**Method**	Available routines, standardised working methods, regulations, instructions	Lack of routines, working methods, instructions
**Result**	Expected results, predictable outcomes	Expected results, unpredictable outcomes

Furthermore, this step included identification of challenges, uncertainties or tensions that had an impact on the work practices and different stakeholders’ perspectives, and where different needs may occur.

The *third step* was to map the critical work practices in relation to ‘Improvisational handling types (A, B and C)’, by assessing the discretion level of task, method and result, and thereby associating them to different types of learning situations.

In the ‘Findings’ section of this article below, data were interpreted using the conceptual framework, Tables 1 and 3 as a lens for the analysis. Quotes have served to illustrate the lines of reasoning and to give a rich image of the aspects or phenomena being discussed.

## Findings

A number of examples of critical work practices aimed at handling the COVID-19 pandemic were identified within the studied organisations. The list presented in this section of the article is not exhaustive. Rather, it contains central and critical responses brought up by key informants from the sampled organisations representing the public sector, private sector and intermediaries for these sectors. An important overall observation from the empirical work is that organisations dealt with both upscaling and downscaling work practices triggered by events in early phases of the COVID-19 pandemic. A common approach in studying work practices is the study of scaling up with respect to new products, processes or services that are introduced and implemented. It is, however, obvious that the pandemic has created an unwanted situation in which upscaling is concurrent with downscaling work practices. Consequently, organisations improvise and have possibilities to learn from these critical work practices. This involves different forms of activities ranging from rather small-scale adaptations to more creative-oriented and developmental work.

### Critical work practices – scaling up and scaling down

Several critical work practices were identified and related to their purpose or impact on the organisation, that is, scaling up or scaling down, respectively. Some of them implied a combination of scaling up and scaling down. As previously described earlier, it was imperative to urgently scale up an existing process or develop a new process or alternatively extensively scale down or cease with an existing process. Examples of how critical work practices triggered and initiated tasks in different domains within private, public and intermediary organisations are further illustrated in Tables 4 to 6.

**Table 4.** Critical work practices categorised as improvisational handling type A – rule-oriented learning.

Critical work practices		Illustrative examples
Risk analyses [↑]	Task	- Risk analyses related to the impact of COVID-19 from several perspectives
	Method	- Performing systematic risk analyses with added focus on COVID-19 based on different topics and levels: society, organisation, customers and clients - Merging different risk analyses through cross-border collaboration - Creating possibilities for digital risk analyses and software development
	Result (expected)	- Action plans - Proactive counter measures
Work environment approaches [↑]	Task	- Securing a safe work environment for employees, customers, society - Systematically approaching work environment issues
	Method	- Rearranging workplaces. Providing own transportations for employees. - Cross sector cooperation to provide necessary protective equipment - Testing new approaches, cross-function risk analysis, upgrade of work environment work practices
	Result (expected)	- Proactive measures. Healthy and safe workplaces (on and off site) - Access to necessary personal protective equipment - Preventing spread of infection
Communication, information [↑]	Task	- Internal and external information regarding policies, approaches and applications related to the pandemic
	Method	- Adapting communication and information strategies - Adapting channels and digital solutions - Cross-border communication and information sharing
	Result (expected)	- Transparency within the organisation - Employee access to relevant information
Short-term furlough [↓]	Task	- Quick adaption to lower demand of work force in own organisation due to decreasing business
	Method	- Investigation of authorities’ offered possibilities and following strict national regulations - Decision of the extent of short-term furlough for individuals’ work, forecasting and scenario-based planning - Replanning work tasks during lower demand - Driving and initiating political suggestions to authorities
	Result (expected)	- Adaption of work force - Survival of business through crisis

**Table 5.** Critical work practices categorised as improvisational handling type B – goal-oriented learning.

Critical work practices		Illustrative examples
Remote work [↑]	Task	- Immediate transition to working from home for possible work tasks, activities and professionals - Adaption to and interpretation of new changes related to authorities’ regulations and recommendations
	Method	- Distribution of ‘working at site’, ‘working from home’ and ‘hybrid work’, and arrangements of new workplaces - Digital tool solutions, securement of the General Data Protection Regulation (GDPR) in digital solutions - Development of policies ‘working from distance’ and new work procedures
	Result (expected)	- Adaption related to swift changes with radical change of working conditions, both for employees working from home and on site
Crisis management [↑]	Task	- Developing a crisis management process related to the COVID-19 pandemic - Developing own organisations’ regulations and policy (national and local)
	Method	- Mobilisation and reprioritisation. Entering staff mode. - Further development of new crisis management organisation - Development of leadership support - New cross-unit collaboration in analysis and forecasting work - Regular meetings and exchange with authorities. Continuously adapting internal approaches to changes in restrictions
	Result (expected)	- Strategic supportive approaches throughout the organisation - Proactive and reactive counter measures
Production capacity, staffing planning [↓]	Task	- Mobilisation within organisations and adapting to fast change in staff needs - Rapidly managing the need of work tasks
	Method	- Developing and coordinating staffing plans across traditional borders - Widening work areas for employees – local, region, national – by utilisation of digital solutions
	Result (expected)	- Meeting demands of increased and decreased needs of work force - Flexible staff
Competence, reskilling, training, education [↕]	Task	- Reskilling activities for employees working in organisations with radically decreased demand due to the pandemic
	Method	- Rapid training and education activities towards reskilling preparing employees for new tasks at other workplaces and sectors - Development of structured digital training in digital tools - Employees learning from each other in daily work - Sharing knowledge within and beyond own organisation
	Result (expected)	- Supply of competences - Supporting society with workforce needed in challenged workplaces - Offering new job opportunities for employees - Retaining performance and efficiency during remote work
Securing the supply chain [↑]	Task	- Securing material supply – finding new suppliers among local, regional or national companies
	Method	- Cooperation in society, sharing equipment between companies and organisations in new ways
	Result (expected)	- Supply of material and production equipment - New strategies minimising risks in the supply chain
Transformation of products, services [↕]	Task	- Production of protective equipment (visors, masks, aprons, barriers, etc.) - Developing new customer offers (education, training)
	Method	- Transition in production from core products to new products - Adapting facilities and premises. New CE (conformité européenne) certification of production lines. - New cross-organisational collaboration developing new services and offers
	Result (expected)	- Supporting society needs. Supply of protective equipment - Strengthening the business, finding new offers

**Table 6.** Critical work practices categorised as improvisational handling type C – creative-oriented learning.

Critical work practices		Illustrative examples
New business areas [↑]	Task	- Developing business towards new market and customers
	Method	- Finding new ways of maintaining turnover, broader perspectives to look at possible new markets in other sectors - Developing digital solutions
	Result (expected)	- New business and markets through digital transformation and solutions, green transformation of business due to increased climate awareness

#### Scaling up

The study identified scaling up work practices such as rapidly switching to remote work, risk analyses, work environment approaches, securing the supply chain, transforming production and services, and finding new businesses areas.

Within both the private and public sector, there were examples of scaling up work practices due to material shortages. One example was the task of a company to rapidly find new local suppliers for their businesses and production as there were limitations in supply chains caused by lockdowns and deficiencies in ordinary structures and processes. Other scaling up examples were collaboration due to the urgent need to support employees in exposed sectors and organisations and find new jobs in the region. As the government and authorities developed new recommendations and legal requirements, management needed to be alert and adapt to this in their own organisations with short notice and quickly offer new mobility solutions for their employees. However, these new demands had a high impact on working conditions not only in the direct work related to health care, but also among professionals in other complementary functions such as laboratory personnel.

#### Scaling down

The study identified work practices scaling down such as staffing in market-exposed sectors, an adaptation of staffing needs by short-term furloughs, training ceased regarding practical elements, for example, training regarding operating forklift, traverse, assembly and welding. Furthermore, there were examples where improvement and development work were cut down, as well as other investigative work. Public and private sectors with radically changed demand such as in aviation, transportation and culture industry were subjected to rapid scale down of personnel, also with impact on surrounding staff working with bookings, conference facilities, light and sound, rental offers, etc.

#### Scaling up and scaling down

The study identified examples of work practices and actions where organisations in parallel scaled up and scaled down, such as production capacity, crisis management, reconfiguring organisations, reprioritisation of education areas, the transformation of production converting production to new types of products, etc.

An example from intermediary organisations illustrates the dynamics between upscaling one part of the organisation while at the same time, downscaling another part. In this case, a white-collar trade union very quickly needed to respond to a government agency’s decision to introduce the possibility for companies to apply for short-time furlough. This was done by mobilising all possible personnel at the trade union to work with negotiations and agreements in relation to the short-term furlough. All other activities such as political case advice, opinion formation, education and communication ceased to meet the increased demand to work with the new agreements:

The inflow [of client requests] increased by 300% from one day to the next. This escalated quickly to 600% during the spring. (Intermediary)

The scaling up of handling agreements included identifying all personnel with any labour law experience and competence to work with negotiations to focus on that. Other personnel were quickly trained to support the union’s locally elected representatives within new areas. Other examples of new cross-organisational collaborations came from public education sectors, when cultural education and laboratory exercise periods at schools were scaled down, and staff rapidly shifted their work to contribute to other educational areas, where the demands increased due to the pandemic.

### Improvisational handling – examples of tasks, methods and results

The respondents presented several tasks, methods and expected results related to the critical work practices categorised into different types of improvisational handling (Tables 4 to 6). In the tables, critical work practices that were scaled up are marked with [↑], those that were scaled down with [↓], and if both scaling up and scaling down were identified marked with [↕].

#### Improvisational handling type A – rule-oriented learning

Findings of critical work practices that were categorised as improvisational handling type A and that were characterised by rule-oriented learning are summarised in Table 4.

Regarding these illustrative examples of critical work practices, the tasks were given. Methods were primarily given, but improvisation took place when adapting and combining existing methods in new ways. There was a great deal of uncertainty regarding the outcome.

##### Risk analyses

In all organisations, there was an immediate need to carry out risk analyses related to the pandemic. Methods used were new approaches combining existing risk analyses that simultaneously addressed different perspectives, for example, the impact of the pandemic on their businesses, stakeholders, employees and suppliers. In the public sector, there were examples of combined ‘patient safety rounds’ and employees’ ‘work environment assessment rounds’, involving more groups in the analyses. By these approaches, they could address different needs and potential contradicting demands from different stakeholders’ perspectives and challenges in new ways, resulting in new solutions that were beneficial for more parties. Uncertainties and challenges were mainly related to the lack of know-how in society of the impact of the pandemic and relevant countermeasures, and how to consider many influencing factors. Experiences from new ways of working with risk analyses in new constellations, initiated reflections of development opportunities, for example, improvements in work procedures with broader cross-border collaboration, close manager and employee participation, and software tools for risk analyses.

##### Work environment approaches

In many organisations, there was a sense of urgency leading to extraordinary attention on the work environment. Tasks were initiated to systematically approach work environment issues securing a safe work environment. Besides finding new solutions to provide employees with necessary protective equipment (and prevent shortages), workplaces were re-designed and reorganised. Creative solutions were implemented, such as dedicated bus transports for personnel from contact points in cities to the production facilities to avoid a spread of infection by public transport. The variety of individuals’ work situations, health risks related to COVID-19 at workplaces, and managing different prerequisites was challenging, as well as uncertainties about the impact of the countermeasures. There were examples of reorganising the work environment, setting aside previously established work procedures. The expected results of these initiatives were prevention of health risks by proactive measures, access to necessary personal protective equipment and enabling healthy and safe workplaces for all employees. Promoting factors, reflected on as ‘lessons to be learned’ post-pandemic, were for example the benefits of close and frequent collaboration between management, employees from different workplaces and trade union representatives.

##### Communication, information

High attention was given to tasks related to information and communication efforts. Organisations were dependent on external information from authorities, and so on, information which could rapidly be changed. Uncertainties were also related to how information was perceived among employees and how middle management continued to convey information. In addition to established methods, there were several examples from all sectors of taking new initiatives for improvements of communication strategies and reorganising information infrastructures for information dissemination and communication. Several organisations started regular digital company meetings, gathering the whole organisation virtually for company updates where management provided information to employees and offered an opportunity for dialogue and questions. Apart from dealing with information related to the pandemic, an effect of increased dialogue was that other issues were raised, which widened the employees’ perspectives:

That’s been really, really great . . . a lot of people haven’t seen these people or ever come close to these issues, and that’s spread awareness. (Private sector)

Furthermore, a common challenge in private sector organisations was possibilities for employees working close to production to receive information. For example, one company invested in iPads for all blue-collar workers to enable efficient communication, information and transparency. Hence, many aspects came to light during the crisis, where structures for management support and professional communication strategies were considered strong enablers for handling challenges and creative solutions.

##### Short-term furlough

Market-exposed organisations needed to rapidly adapt to new conditions, requiring challenges regarding staffing. A critical and clearly expressed task was to maintain personnel during the period of lower market demand and quickly adapt to new conditions. Many organisations had the opportunity to achieve support as Swedish authorities offered a short-term furlough programme. These support programmes were strictly regulated and needed to follow a certain procedure. Intermediaries, such as trade unions on a national level, reallocated their work tasks with assignments to support and manage new types of problems that arose from organisations and jointly discuss possible solutions with employees at different workplaces, as expressed:

We had to re-prioritize . . . and promptly train all personnel to support our elected representatives in how to deal with the current situation. (Intermediary)

At some workplaces in the private sector subjected to short-term furloughs, there were uncertainties in fast-shifting market demands and challenges on how to adapt to the changes and to reorganise in the new upcoming situations. Specifically, how to manage the work processes, deliverables and tense working conditions with radically changed prerequisites in a fast-changing environment. Furthermore, the sensitive work situation led to discussions about how down-turn periods in future could be utilised for training and competence development, which could enable flexibility and responsiveness towards fluctuations.

Regarding these critical work practices, the tasks were thus given. Methods were primarily given, but improvisation took place when adapting and combining existing methods in new ways. There was a great deal of uncertainty regarding the outcome.

#### Improvisational handling type B – goal-oriented learning

Examples of critical work practices of improvisational handling type B and characterised by goal-oriented learning are summarised in Table 5. Here, two out of three of the aspects task, method or result were ‘not given’. The tasks were given, but these work situations required further development of both existing methods as well as improvisation to find new approaches that were not applied or tested before. The expected results were not given beforehand.

##### Remote work

Work from home was required for professionals with possibilities of carrying out their work tasks remotely, to reduce the risk of infection based on authorities’ and their own organisation’s regulations and recommendations. Methods used were immediate transition with an uncertainty of the outcome, both for individual and organisational performance. Examples were distribution of appropriate work tasks ‘from site’ or home respectively, digital tool solutions, training in digital tools and digital facilitation, and securement of data management. There were uncertainties regarding the eventual consequences on strategic work, performance and efficiency, collaborations, relationships and networks, leadership, and the business itself. Challenges addressed were developing digital communication platforms, information security, solutions for creative meetings, competence development activities and workshops, information technology (IT) support and safety routines in organisations, which they were not prepared for. Furthermore, a learning potential was discussed regarding benefits for recruitment and supply of competence, for example, remote work offering new opportunities for attracting talent, and how these experiences would have an impact in the long term, in a young, future work generation’s mind-set and work mobility desires. In addition, the rapid transition to utilising digital tools initiated reflections on the development of future work with increased possibilities for work flexibility for both white-collar and blue-collar work, which is beneficial for both individuals and organisations. Regarding the latter, there were reflections on the use of work premises and how decreased travel could contribute to climate goals and the development of new green businesses.

##### Crisis management

In general, management tasks were to further develop strategy and action plans based on the authorities’ recommendations and restrictions. Methods were for example entering staff mode, mobilisation, a transformation of focus to develop a crisis management process, increased internal reconciliation meetings, and new cross-border collaborations with authorities. Organisational changes were identified, such as creating an extra organisational level to increase the ability of rapid decision-making. There were several examples of new management approaches during the crisis characterised by clear decision-making with ‘authoritarian leadership combined with trust-based leadership’ expressed as,

You step into an operational mode but with very clear assignments . . . we staffed up a specific management support organisation . . . we dealt with it as a state of emergency. (Public sector)

Uncertainties were related to business survival, performance and how the pandemic would develop. Furthermore, there were challenges such as balancing stakeholders’ needs and competing requirements, setting priorities, actions constantly mapped and related to swift changes, high pressure, lack of experience and proactive approaches towards crisis. Discussions were initiated on how to understand and approach stakeholders’ different needs, multiple tensions and develop resilient organisations, for example, how to learn from the crisis, improve and make better priorities, and composition of crisis groups.

##### Production capacity – staffing planning

Swift changes of production capacity requirements required tasks related to staff planning. For example, within the public sector, an important task was to provide production capacity in terms of number of ‘health care beds’. At these workplaces, the tasks were clearly expressed, but with uncertainty about the actual needs, both regarding amount and type of health care. During this period, there were major actions in building temporary hospitals such as tents. The expected results from this mobilisation were increased health care capacity, but where there were estimated goals of the actual needs of temporary hospital facilities and staff. From a staffing planning need, methods used were coordinating staffing plans across traditional borders, as well as alternative staffing plans, due to the high uncertainty. Staff mobility was needed across organisational borders (internal, external), where digital solutions also provided possibilities for widened work areas for employees – local, regional, national. Other examples in the public sector were possibilities for dental nurses reassigned to completely different services, flight attendants retrained to assist nurses, and so on. Although there were contingency plans and previous work procedures to rely on when adapting to a variety of demands, the rapid need for increased capacity dealing with a new disease required the initiation of several new innovative approaches. Based on these experiences, long-term effects and new possible opportunities were discussed regarding mobility and flexibility working across borders and how that could be perceived among customers, existing staff and among forthcoming young new work generations.

##### Competence, reskilling, training, education

Several tasks such as reskilling of employees needed to be carried out among employees at market-exposed workplaces. Methods used for increasing skills and competences were increased knowledge sharing between individuals (everybody learning from each other in their daily work), development of structured training and increased benchmarking. Apart from supporting society with the necessary skills and workforce at challenged sectors and workplaces, the expected results were to offer new job opportunities for employees at market-exposed workplaces during the time of the crisis. However, there were challenges related to time pressure to train new personnel, the influence of stress and anxiety of the impact on job opportunities. Reflections of learning beyond the pandemic were related to further developing structures and opportunities for competence development enabling work flexibility across borders.

##### Securing the supply chain

Urgent tasks in private companies were to find new suppliers among local, regional or national companies. Challenges and uncertainties were fluctuations of material supply and the pandemic impact globally, in the entire supply chain. Methods used were new cross-collaborations across traditional borders. For example, within public sector organisations, there were regional initiatives building a central warehouse planning to support several organisations within municipalities with protective equipment. In private sector organisations, the issues were raised to the top management level to both manage the actual situation as well as to set up new strategies for securing the supply chain. A surprising insight was how technical resources such as existing equipment in industrial companies could be utilised for other purposes within health care in a municipality:

We are blurring the boundaries now, we almost have no boundaries, but we can help and support each other in the region, the one who has the time, skills and space can take that client. (Intermediary)

Furthermore, these challenges had impact on the awareness of organisations’ vulnerability to disturbances and external dependencies. Hence, learning was discussed regarding new strategies for securing the supply chain and possibilities to develop new local/regional solutions.

##### Transformation of products, services

In both sectors, tasks were clearly given to transform products and services due to the COVID-19 pandemic. There were industrial companies in the private sector shifting their production to produce hand sanitiser and protective equipment (visors, masks, aprons, barriers, etc.). There were several approaches and industrial methods enabling this transition. New partnerships and stakeholder collaborations were initiated in society, with examples of sharing equipment between companies and organisations. Also, in the public sector, there were examples of rapid transition of customer offers to new services and new customer groups. One of those was within a municipality that developed training for swimming skills for new age groups and initiated new cross-border activities between cultural and education sub-sectors within the municipality, which resulted in positive effects for learning skills in society as well as benefits for employees and organisations. There were uncertainties related to customer perception of the new offers and swift changes in market demands, and challenges were for example external dependencies on the authorities’ decision process for required approvals and their crisis preparedness. However, these challenges had an impact on the development of completely new offers such as new education concepts, new machine investments and recruitment. Furthermore, learning potential was discussed regarding completely new possibilities due to the rapid digital transformation, flexible customer offers and benefits of working cross traditional borders.

#### Improvisational handling type C – creative-oriented learning

Findings of critical work practices with respect to the aspects task, method and expected result, and characterised by creative-oriented learning are summarised in Table 6.

For improvisational handling type C, the tasks related to critical work practices were not clearly defined, and neither the methods given to approach this identified need nor the results were possible to predict. Thus, this required a high degree of creativity and improvisation.

##### New business areas

Many industrial companies lost their core market from 1 day to the next, needing to initiate tasks to rapidly find new ways to maintain turnover when production volumes were down. However, there were uncertainties related to the future development of individuals’ behaviours and future markets, that is, regarding climate awareness and transportation. The pandemic required new innovative methods and approaches at a fast pace to find new customers and use digital solutions in these work processes. For example, there were reflections on required business development within the automotive sector. A surprising effect of the pandemic was expressed regarding how small and medium-sized companies rapidly managed transformation to find new markets, where the pandemic crisis triggered the development of new business models, requiring extensive changes in organisation and production. From a business perspective, this was regarded as beneficial in a long-term perspective, as some companies within the private sector were vulnerable and highly exposed to the competition when having few major customers, or a market within the same sector, for example, suppliers within the automotive industry. Possible new business opportunities were discussed regarding the long-term impact of the pandemic on sectors from a society and climate perspective. For example, it was also expressed that ‘the pandemic will be a driver towards a better planet’ that would push transition towards sustainable development of products and production processes forward at a faster pace. There was also reasoning that there probably would be a paradigm shift on human mobility with a strong impact on sectors within the transportation and automotive industry. Presumably, there could be drivers for new business models for these sectors with a long-term positive climate impact.

## Discussion

The aim of this article was to explore the improvisational handling of scaling up and scaling down of critical work practices during the COVID-19 pandemic and interpret these practices from an organisational learning perspective. The study in the early phases of the pandemic showed that a number of critical work practices were initiated in organisations scaling up or scaling down, which required improvisational handling. This comprised the way that organisations tried out, tested and changed their business, working methods and processes during the journey to find solutions. As the organisations were subjected to a high degree of uncertainty, that is, exposed to a revolutionary crisis, the focus was to study the occurrence of improvisational handling and opportunities for learning.

The pandemic has had severe consequences for many organisations. Although we have witnessed many organisations suffering and eventually being forced to close their businesses, there are others that have managed to handle severe difficulties. Some organisations have required an extremely rapid acceleration, that is, scaling up. At the same time, other operations have had to shift sharply and put on the emergency brake, with major consequences for both employees’ working conditions and the operations (cf. Wallo et al., 2012). There are also examples of workplaces within the same organisation that have been heavily mobilised with consequences for the work environment where some parts had to accelerate, while other parts scaled down. In principle, all activities in different organisations have needed to gather strength, re-prioritise, mobilise and change. As a result, private and public organisations as well as intermediaries have suddenly needed to act quickly and adjust to completely new situations, regarding the market, business, organisation, management, competences and societal changes. The results of this study show that in the initial phase of the COVID-19 pandemic, new areas emerged extremely quickly in organisations, areas in which work practices have been scaling up and/or down. Within these work practices, tasks have been initiated to solve problems, methods and approaches have been modified, and there have been variations in the predictability of the results, such as effect and types of outcomes of these efforts. The scaling up and down of work practices has thus been an important basis for organisational improvisation.

**Figure 1.**
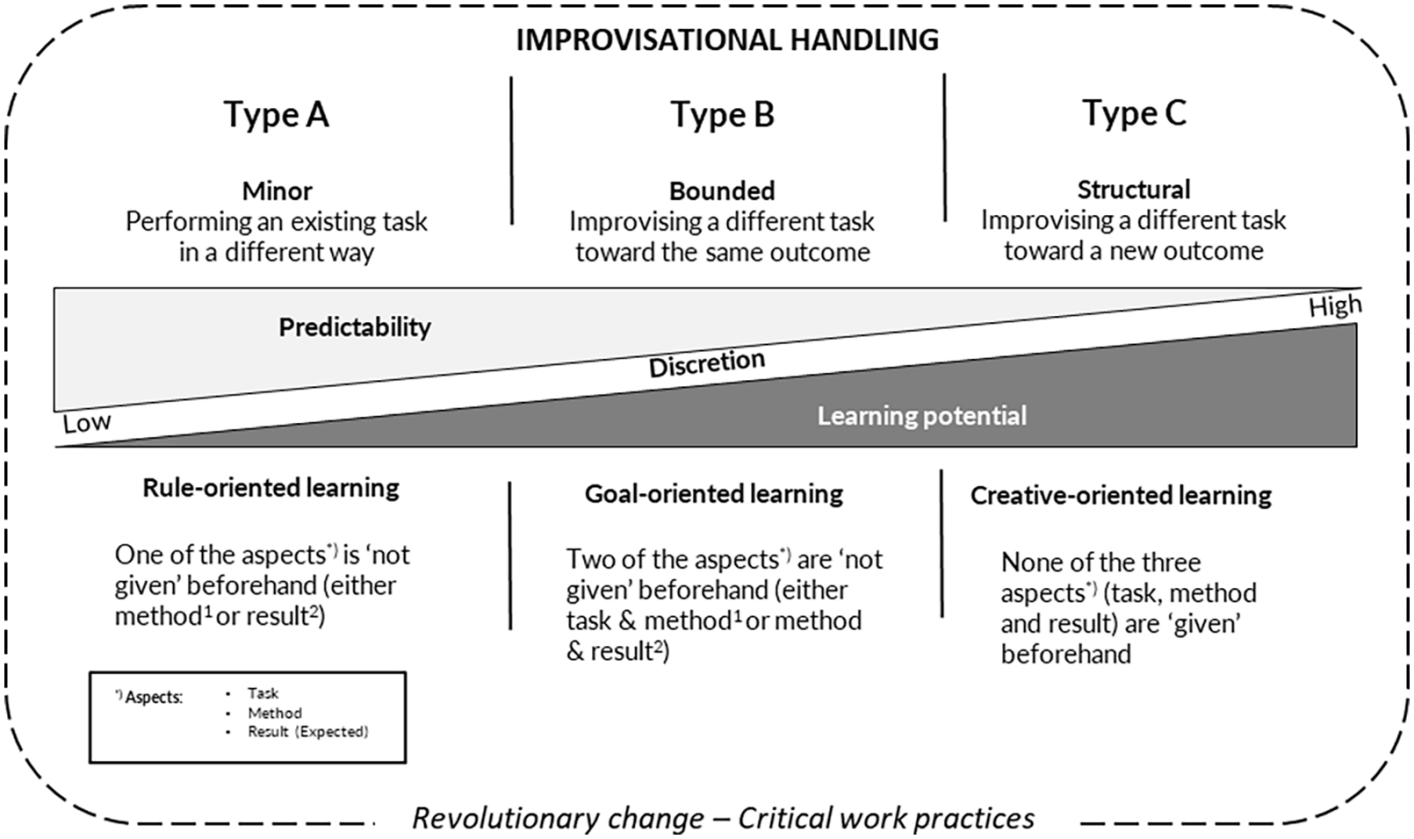
Model for improvisational handling in relation to degree of improvisation and level of learning. Adapted from Hadida et al. (2015) and Ellström (2001, 2006).

The results of this study further show that these improvisations varied in degree of improvisation (Hadida et al., 2015), from minor (Improvisational handling type A) to bounded (Improvisational handling type B) and in some cases on towards structural improvisation (Improvisational handling type C). In this article, the degree of improvisation has been linked to the discretion in task, method and expected result, described by Ellström (2001, 2006) as shaping the levels of learning that exist in work situations. By combining these two frameworks, a model for degree of improvisation and level of learning has been put forth, in which the discretion of tasks, methods and expected results increases along a continuum from minor, bounded to structural improvisation, while at the same time, the certainty and predictability decrease. Looking at the learning potential in parallel, it increases along with expanded discretion of task, method and expected results (see Figure 1).

The findings identify challenges concerning learning opportunities during the crisis in the initial phases of the pandemic. These challenges encompassed tensions based on different needs or contradictory demands that may occur, hence needed to be considered during fast-paced changes in organisations (Meisiek and Stanway, 2022; Smith and Lewis, 2011). One identified tension was the employees’ different exposures to health risks due to COVID-19 at workplaces. To decrease risk exposure, large groups of employees were transferred to work from home, while other employees had work tasks that needed to be carried out physically near customers, clients, services or the production process. There were also examples of how official regulations were interpreted differently at workplaces causing varying working conditions and risk exposure for employees. During the initial phases of the pandemic, the findings indicate that overall decisions were carried out on a (crisis) management level, with need for rapid distributed decision-making within organisations and locally at workplaces. Thus, there was potential risk for lack of understanding of perception and impact of the tasks and approaches from an employee perspective, and furthermore risk of lack of awareness of needs and eventual conflicting demands from a systems perspective.

There were also challenges regarding individual competence development opportunities during the crisis. The employees’ learning opportunities in daily work accelerated when rapidly managing new problems and uncertainties, while formal training activities and strategic competence development initiatives were either cancelled or shifted to digital solutions. Those training activities that were cancelled primarily involved training needed to be performed physically at site. There were also different opportunities for access to formal training activities for employees who needed to be on site during work, compared with employees working from home who could participate in digital education activities and development activities sharing experiences. This distinction between different groups of employees also involved different opportunities for access to information and communication through digital channels. Thus, tension could occur due to perceived injustice between employees’ opportunities for formal competence development as well as possibilities for sharing experiences, which is a base for organisational learning (Crossan et al., 1999). The findings show that during the improvisational handling of the pandemic, there is increased discretion when performing work, which leads to an increased potential of individual learning. However, if the individual employees do not share and reflect on these experiences, there is a risk that the learning will remain on an individual level and not reach the goal of organisational learning.

A reflection from these findings is that tensions may have an impact on the learning potential during improvisational handling as they may cause segregation between employees, become barriers to organisational learning while dealing with challenges and new problems during fast changes, and lead to an undesired development of the organisation. Similar findings from other studies during the pandemic are addressed by Meisiek and Stanway (2022), who emphasise the means of power relations, that relations between individuals and groups have an impact on learning during a crisis, and that these aspects need to be taken into account to enable learning from a long-term perspective. During crisis requiring improvisational handling in an organisation, there is a high potential for individual learning and increased discretion performing tasks. The opportunities for learning and transformation during dramatic changes are also found in other research as reported by Karjalainen et al. (2022). Another reflection from our findings is the means of involvement, trustful relations, information and communication that influence the expected results and desired outcomes. Hence, the need to connect values and social dynamics to organisational improvisation is crucial, as stressed by Hadjimichael and Tsoukas (2022). From both the individual and the organisational perspective, it is important to achieve equality regarding access to information, collaboration and learning opportunities, and avoid risks of developing sub-optimal solutions, not sustainable over time. Furthermore, the findings indicate managerial challenges of combining informal and formal learning opportunities when employees’ working situation radically changes as different needs or contradictions may arise. Thus, in line with Smith and Lewis (2011), it is increasingly important to reflect on individuals’ and stakeholders’ different needs and conflicting demands during fast changes in an increasingly complex reality, and to continually integrate these aspects in management strategies during improvisational handling.

Hadida et al. (2015) further distinguish between different levels of improvisation, referring to whether the improvisation takes place within an individual, between a few employees, or organisationally. This is similar to the framework of organisational learning that highlights how learning takes place in a complex interplay between the individual, group and organisational levels (Crossan et al., 1999). In our study, we have found different examples of individuals’ learning during the COVID-19 pandemic and that this learning has been used further in their work. Work practices have been changed and developed. Although this has comprised actions guided by intuition (cf. Crossan and Sorrenti, 1997), these actions have been made use of collectively and new routines have been developed at the organisational level (Crossan et al., 1999). This has been beneficial in later phases of the pandemic crisis, thus constituting an indicator of organisational learning. The employees’ work environment has also been seen in a new light during the pandemic crisis, where investments in the individual’s working conditions, new forms of collaboration, use of digital aids and competence development have been prioritised, with many innovative elements as a result. Furthermore, already early in the pandemic, temporary solutions were introduced, some of which were considered to perhaps become permanent and institutionalised as organisational learning (Crossan et al., 1999; Wallo et al., 2012). One important result was the potential and strength when combining previously established and new working methods. The study thus shows that the improvisations during scaling up and down processes during the pandemic contribute to organisational learning.

With respect to the identified Improvisational handling types A, B and C, there were different conditions related to the tasks, triggered by the critical work practices and methods used to achieve the desired outcome, thus offering opportunities for learning (Ellström, 2001, 2006). First, improvisational handling type A was found in all interview groups and carried out in critical work practices with an impact on organisations’ scaling up and scaling down. In both cases, the tasks were clear to handle explicit problems, but with an urgent need of transition in organisations. In the scaling up example of ‘risk analyses’, there were given methods for individual risk analyses addressing certain areas, but commonly, there were no given methods for cross-functional collaboration merging these risk analyses and jointly finding new solutions related to COVID-19. Furthermore, as the results were not given beforehand, there was a need for awareness of potential risks both from the individual’s and the organisation’s perspective. In the scaling down example called ‘short-term furloughs’, completely novel conditions arose, requiring improvisational handling regarding how to realise the tasks in a completely new situation with new challenges. Indicators of rule-oriented learning (cf. Ellström, 2001, 2006) were that organisations during the crisis continuously introduced new routines, policies and so on, adapting to the new working situation. As only one out of three aspects was not given beforehand, these were considered minor improvisations (cf. Hadida et al., 2015).

Second, improvisational handling type B was the most common type of learning situation identified in the study and also observed in all interview groups, as tasks were performed in a context where complex problems needed to be managed. The tasks required improvisational handling to a higher degree than type A and were therefore considered bounded (cf. Hadida et al., 2015), and individuals faced situations where predetermined routines could not be applied or relied upon. By taking steps, testing, applying and evaluating steps, the learning situations were characterised by goal-oriented learning. From an organisational learning perspective (Crossan et al., 1999), insights were drawn during the scaling-up example into remote work, where several advantages but also new types of challenges were highlighted. The crisis resulted in a rapid development of digital skills among the existing workforce. This can also be fruitful in the long term as it increases the preparedness among the current workforce and organisations to meet new overall demands on competitiveness. This includes meeting forthcoming new generations of employees with existing skills and work expectations within this area.

Third, the study identified major critical work practices, addressing complex challenges requiring tasks for finding new markets and business models to survive the crisis. However, it was expressed that the pandemic stressed desirable mega-transitions in industrial sectors that are necessary for sustainable development from a climate perspective, requiring reconsideration of existing core business areas. In this example, neither the tasks, methods nor expected results were given beforehand, and thus required a high degree of innovativeness and breaking norms. This type of handling, described as improvisational handling type C, is structural (Hadida et al., 2015) and provides opportunities for creative learning. Thus, the study shows that the pandemic has involved situations that required different types of improvisational handling in a context of uncertainty, which enabled a higher degree of expansive learning, compared to the degree of adaptive learning (Ellström, 2001, 2006).

The group of intermediaries included in our study had a dual perspective by contributing experiences from their own organisations during the pandemic but also with an overarching view on consequences for the sector they represented. Having a central role within the Swedish labour market, they could forward information and be a node for the exchange of experiences between organisations. Findings from the study showed that the national restrictions and recommendations increased the importance of the intermediaries’ function and role between authorities and organisations. Apart from rapidly supporting individual organisations in negotiations related to central agreements regarding short-term layoffs, they served as a link for cross-organisational learning between workplaces and organisations through several networks that they coordinated.

During the pandemic, many organisations have joined forces to deal with the crisis. That has been necessary due to limited resources, which is one characteristic of improvisation as drawing on available resources (Cunha et al., 1999). The pandemic is thus an example of resource constraints and suspension of established practices causing a disruption, which has been observed regarding how we work and live (Orlikowski and Scott, 2021). However, this has also been identified as a means to motivate individuals to improvise within venture development and entrepreneurship (Baker and Nelson, 2005).

Overall, the pandemic has put stress on both individuals and organisations. The radical change of working conditions has raised issues needed to be handled in organisations, such as work environment, motivation, self-management and trust-based leadership, as well as the boundaries between individuals, colleagues, management and external parties such as customers and suppliers. For example, if remote work would continue, the organisations will need to develop further to meet new potential challenges related to collaboration, performance, health, well-being and so on. Furthermore, the pandemic has challenged the organisational processes by visualising what is important and what works (or not). This has been a driver for action, change and development. The study also has shown that the pandemic has released established structures in organisations, as they have had to manage many new types of emerging problems that urgently needed to be solved. The improvisational handling in the scaling up or down of the critical work practices has increased the discretion in tasks, methods and expected results and thereby implied increased room for manoeuvre. This in its turn has resulted in learning at individual, group and organisational levels, which may strengthen the organisations’ ability to meet future unprecedented challenging situations.

## Conclusions, limitations and implications

In conclusion, this study has shown that the improvisational handling of critical work practices during the pandemic resulted in some temporary solutions, tasks, methods and results that may become implemented and established after the pandemic. Due to uncertainty and emergency of the improvisational handling, both individuals and organisations were challenged, individuals in terms of demanding changes of work situations and organisations in terms of the emergent need to scale critical work practices up and/or down with consequences for other operations. The uncertainty and emergency along with loosened boundaries of established structures have increased the space of action. The study shows that the COVID-19 pandemic has served as an enabler for improvisation, which has resulted in changes. Some of these changes may have been discussed in the organisations before the pandemic but during the crisis they have been brought to the fore, forcefully acted upon and overcome previous limiting structures (Crossan and Sorrenti, 1997).

The study further shows that improvisational handling during the pandemic has also resulted in learning at different levels and thus can be regarded as a source for organisational learning. With a view to the future, a question arises whether the organisations through the pandemic have learnt how to improvise. This is a skill that can be learned (Crossan and Sorrenti, 1997) and a key part of a crucial competence that is needed to increase the organisation’s preparedness for future dramatic changes and crises. In this regard, more research is needed to understand how critical scaling up and scaling down of work practices, related to radical societal challenges such as the COVID-19 pandemic, affect the potential for improvisational handling and opportunities for organisational learning during increased complexity in fast-moving work environments. To overcome the limitations of this study stemming from the cross-sectional design and the use of self-reported data that may contain discrepancies between what people say they do and what they actually do, studies following work practices over time are needed to identify the mechanisms that facilitate organisational learning in times of crisis. With a longitudinal, mixed-method design, it would be possible to gain statistical evidence linking improvisational handling with various organisational learning outcomes, while also allowing for a more in-depth analysis of how the daily, micro-oriented practices and routines are affected.

Concerning implications for practice, the proposed model on improvisational handling in relation to the degree of improvisation and levels of learning can be used as an analytical tool for managers to highlight, analyse and learn from the changes in work practices that were set in motion during the pandemic. Such learning may improve the ability to cope with future rapid and creeping crises. The model may also be used in management education to encourage critical reflection on how organisational agility and resilience can be facilitated by processes of improvisation and developmental learning during situations with a high degree of external change pressure.

## Notes

1. https://www.government.se/articles/2020/04/strategy-in-response-to-the-covid-19-pandemic/ (Published 9 November 2020. Accessed 8 November 2022).

2. https://www.folkhalsomyndigheten.se/nyheter-och-press/nyhetsarkiv/2020/mars/personer-over-70-bor-begransa-sociala-kontakter-tills-vidare/ (Published 16 March 2020. Accessed 3 December 2021).
